# Cancer incidence and mortality in the Bucaramanga metropolitan area, 2003-2007

**Published:** 2012-12-30

**Authors:** Claudia Uribe, Sonia Osma, Víctor Herrera

**Affiliations:** a Cancer registry Bucaramanga metropolitan area. E-mail: curibep@unab.edu.co; b Program of medicine, Universidad Autónoma de Bucaramanga, Bucaramanga, Santander, Colombia. E-mail: sosma3@unab.edu.co

**Keywords:** Cancer, incidence, mortality, population-based cancer registry, metropolitan area

## Abstract

**Introduction:**

Cancer is an important cause of morbidity and mortality worldwide. Population-based cancer registries (PBCRs) make possible to estimate the burden of this condition.

**Aim:**

To estimate cancer incidence and mortality rates in the Bucaramanga Metropolitan Area (BMA) during 2003-2007.

**Methods:**

Incident cases of invasive cancer diagnosed during 2003-2007 were identified from the Bucaramanga Metropolitan Area PBCR (BMA-PBCR). Population counts and mortality were obtained from the Colombian National Administrative Department of Statistics (NADS). We estimated total and cancer-specific crude incidence and mortality rates by age group and sex, as well as age-standardized (Segi's world population) incidence (ASIR(W)) and mortality (ASMR(W)) rates. Statistical analyses were conducted using CanReg4 and Stata/IC 10.1.

**Results:**

We identified 8,225 new cases of cancer excluding non-melanoma skin cancer (54.3% among women). Of all cases, 6,943 (84.4%) were verified by microscopy and 669 (8.1%) were detected only by death certificate. ASIR(W) for all invasive cancers was 162.8 per 100,000 women and 177.6 per 100,000 men. Breast, cervix, colorectal, stomach and thyroid were the most common types of cancer in women. In men, the corresponding malignancies were prostate, stomach, colorectal, lung and lymphoma. ASMR(W) was 84.5 per 100,000 person-years in women and 106.2 per 100,000 person-years in men. Breast and stomach cancer ranked first as causes of death in those groups, respectively.

**Conclusion:**

Overall, mortality rates in our region are higher than national estimates possibly due to limited effectiveness of secondary prevention strategies. Our work emphasizes the importance of maintaining high-quality, nationwide PBCRs.

## Abstract (es)

**Introducción:**

El cáncer es una causa significativa de morbi-mortalidad y conocer la magnitud del problema es posible gracias a los Registros poblacionales de cáncer (RPC).

**Objetivo:**

Describir las tasas de incidencia y mortalidad por cáncer en el área metropolitana de Bucaramanga (AMB) durante el quinquenio 2003-2007.

**Metodología:**

Los casos de cáncer invasivo primarios del período 2003-2007 se seleccionaron de la base del Registro poblacional de cáncer del área metropolitana de Bucaramanga (RPC-AMB). Los datos de población y mortalidad se obtuvieron del Departamento Administrativo Nacional de Estadística (DANE). Se estimaron tasas crudas de incidencia (TC) y mortalidad (TMC) totales y específicas por grupos de edad y sexo, y tasas de incidencia (TEE) y mortalidad (TMEE) estandarizadas. Los análisis estadísticos se hicieron con CanReg4 y Stata/IC 10.1.

**Resultados:**

Se reportaron 8,225 casos de cáncer incidentes al RPC-AMB durante el quinquenio (se excluye cáncer de piel no-melanoma), con un 54.4% de casos en mujeres. La TEE global fue de 177.6/100,000 hombres-año y 162.8/100,000 mujeres-año. Los cánceres más frecuentes para la población general fueron mama, próstata, estómago, colon y recto, y cuello uterino. La TMEE estimada en el AMB fue mayor que la cifra nacional, especialmente en los hombres.

**Conclusión:**

Las tasas globales de mortalidad por cáncer en nuestra región son más altas que el estimado nacional, lo que sugiere problemas en medidas de prevención secundaria. Las TEE de cáncer de mama y próstata aumentaron de acuerdo con las tendencias mundiales. Es necesario continuar los esfuerzos de vigilancia del cáncer.

## Introduction

Currently, cancer is one of the major causes of morbidity and mortality worldwide despite the scientific and technological advances that have contributed to improve its diagnosis and treatment. According to the World Health Organization (WHO) in 2008 occurred 12.7 million new cancer cases and 7.6 million cancer deaths in the world^1^. Furthermore, the projected numbers of deaths attributable to cancer are 9.0 and 13.1 million by 2015 and 2030, respectively, an increase that will have the greatest toll on low-income regions.^1^ Globally, in 2008 the most common malignant tumor types were lung cancer (12,7%), breast cancer (10.9%), and colorectal cancer (9.7%) while those causing the highest mortality were lung cancer (18.2%), stomach cancer (9.7%), and liver cancer (9.2%), although both incidence and mortality showed geographic heterogeneity^2^.

In Colombia, cancer is the third leading cause of death and due to its growing impact on population health, the National Cancer Control Plan 2010-2019 established the need to consolidate a national surveillance system supported by already existing population-based cancer registries (PBCRs) such as the Bucaramanga Metropolitan Area PBCR (BMA-PBCR), which started operations in 2000^3^. PBCRs are high quality information systems that guaranty the completeness, accuracy, and comparability of cancer data, essential to inform and guide national public health policies in this matter^4^. The objective of this report is to describe the main indicators of cancer control including crude and age-standardized cancer incidence and mortality rates estimated on the total population and sex-specific groups from the BMA-PBCR during 2003-2007.

## Materials and Methods

We conducted a descriptive study based on the information on invasive cancer cases from the BMA-PBCR during 2003-2007.

### Geographic Area:

The BMA geographic area includes the city of Bucaramanga and three contiguous towns: Floridablanca, Girón and Piedecuesta, all located in the department of Santander, in the Andean region of Colombia, covering an area of 1.479 km2. Although each town is independent in political organization, they are all economically and socially related^5^.

**Figure 1:**
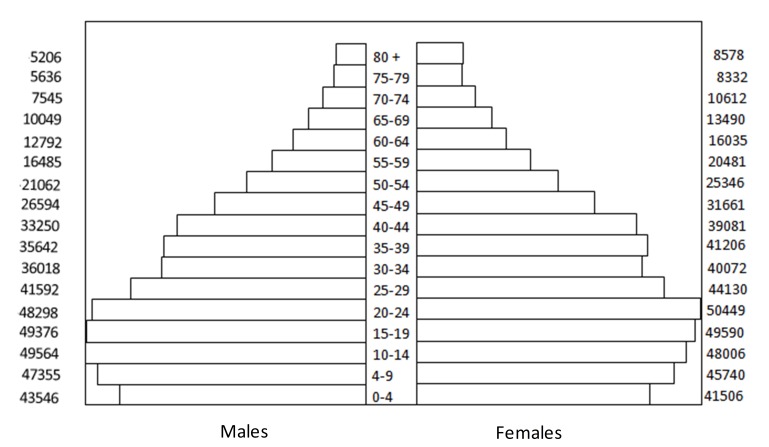
Population structure by age and gender. Bucaramanga Metropolitan Area National Administrative Department of Statistics (NADS)

Currently, the BMA has 1,094,390 inhabitants (taken from the 2005 National Census Projections) corresponding to 53% of the total population of Santander. figure 1 shows the distribution of the BMA population by age and sex. Overall, men constitute the 48% of the population, 26% and 22% of the population is under age 15 and over age 50, respectively, and 94% of the population resides in the urban area. Furthermore, the BMA population is considered racially mixed (high genetic variability) with low socioeconomic differentiation^6^.

From the health care perspective, the BMA has 408 health care institutions including 15 public facilities (hospitals, health centers and intermediate units). These institutions have numerous surgical and chemotherapy units, and some of them have also cancer centers offering radiotherapy services. For this reason, the BMA is considered a referral center for the diagnosis and treatment of cancer patients^7^.

### Bucaramanga Metropolitan Area Population-Based Cancer Registry:

The PBCR started activities at the BMA on April 10th, 2000, as a commitment by the Universidad Autónoma de Bucaramanga (UNAB). Currently, it constitutes a research project funded by grants from the UNAB and the National Cancer Institute of Colombia (NCIC).The BMA-PBCR staff is constituted by a director, a pathologist, a coordinator, three data collectors, and a typist. The registry receives scientific advice and support from a team of professors from different disciplines such as basic sciences, clinical specialties, epidemiology and public health. Furthermore, undergraduate students, who are interested in cancer research, can formulate and develop proposals nested on the registry.

### Data collection and processing:

The BMA-PBCR actively identifies, collects, and registers cancer cases by making regular visits to the primary information sources. In this surveillance system, a case is recordable if: 1) It corresponds to any malignancy located at any site, any central nervous system benign neoplasm, cervical dysplasia or carcinoma in situ; and 2) it was diagnosed after January 1st, 2000, in a patient, urban or rural dwelling, of the BMA regardless of the diagnostic method, including those cases identified by death certificate (excluding patients with basal cell skin carcinoma)^8^.

Registry´s main sources of information were: laboratories of hematology and pathology, hospital discharges, imaging centers, screening centers, volunteer organizations, cancer centers, medical specialists, autopsies and death certificates obtained from the Departmental Health Secretary. The National Administrative and Statistical Department is the government agency that collects, organizes and encodes the underlying causes of death using for this purpose the tenth revision of the International Classification of Diseases (ICD-10), and constitutes the source for mortality^9^ .

Cases obtained from hospital discharges and death certificates were ascertained following a process of diagnosis verification by reviewing clinical histories. In the absence of clinical information, a case was labeled as identified by death certificate only (DCO). Coding of cancer cases was performed by a pathologist with training in the use of the International Classification of Diseases in Oncology, Third Edition (ICD-O-3), and the rules for multiple primary tumors of the International Agency for Research on Cancer (IARC)^10^ ^,^ ^11^.

CanReg^4^ was used for data entry and analysis. This software, designed by the IARC, allows identifying duplication of cases and multiple primary tumors. Data were additionally validated using IARCTools and the Registry PlusTM Link Plus programs. We estimated cancer crude incidence rates using the projected BMA population as denominator. Furthermore, we estimated age-standardized incidence rates (ASIR(W)) and age-standardized mortality rates (ASMR(W)) using direct standardization and Segi's world standard population, corrected by Doll et al.^12^ ^,^ ^13^ . Analysis were conducted using CanReg4 and Stata/IC ^10^ ^.^ ^1^.

### Registry quality indexes:

 Data quality in a cancer registry relies on the quality of both the information sources and the registration process itself. In an evaluation by the INC to the national PBCRs during the period 2000-2005 it was found some degree of underreporting of cases in our registry, partially explained by insufficient data from death certificates. This was a particular situation associated with the Act 079 of 1993, which prevents the National Administrative and Statistical Department releasing death certificate information. Currently the Departmental Health Secretary supports the BMA-PBCR and facilitates access to the mortality database. On the other hand, the BMA-PBCR showed a low index of information inconsistencies in the same evaluation (e.g. duplicated cases, incompatibilities between cancer localization and sex, cancer histologic findings and sex, etc.)^14^.

Finally, the BMA-PBCR complies with the highest standards of confidentiality as recommended by the IACR for the sole purpose of gathering epidemiological data^15^ .

## Results

### Registry quality indexes:

During 2003-2007, 8,225 incident cases of cancer were registered in the BMA among which 4,468 (54.3%) occurred in women. Of all the cases, 6,943 (84.4%) were verified by microscopy (cytology, hematology or pathology) while 669 (8.1%) were detected only by death certificate (Table 1). The proportion of cases verified by microscopy was significantly higher in women compared to men (85.4% versus 83.2%, *p* <0.001) and the opposite was observed for the percentage of cases identified by death certificate only (7.5% versus 8.9%, *p* =0.021). The five types of malignant tumors with the highest percentage of cases verified by microscopy among women were: Melanoma (98.4%), thyroid (97.4%), lymphomas (96.7%), cervix (93.6%) and breast (92.7%). Among men the corresponding tumor types were: breast (100.0%), lymphomas (96.5%), testis (95.7%), melanoma (94.3%) and thyroid (93.2%). Regarding cases detected only by death certificate, locations with higher rates in women and men were: pancreas (37.7% versus 42.9%), liver (27.8% versus 27.1%) and lung (18.4% versus 19.0%).

**Table 1.**
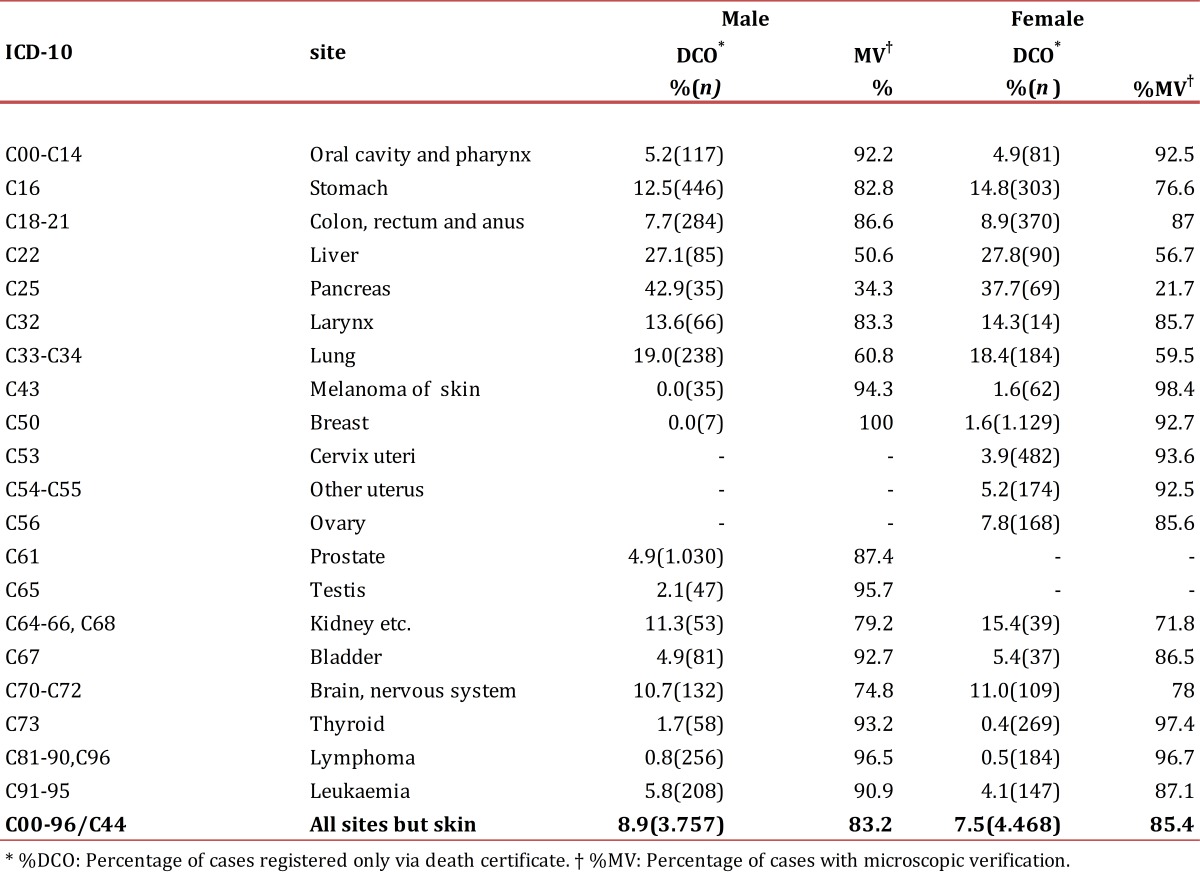
Quality indexes by cancer location and gender. Bucaramanga Metropolitan Area, 2003-2007.

### Relative frequencies:

In the female population the average age at diagnosis of cancer was 58.4 years, and the most common malignant tumors were breast (23.5%), cervix (10.0%), colorectal (7.0%), stomach (6.3 %) and thyroid (5.6%) (Table 2). In men the average age at diagnosis was 62.4 years. Prostate (24.6%), stomach (11.1%), colorectal (6.6%), lung (5.7%) and non-Hodgkin lymphoma (4.4 %) were the most common types of cancer in this group. Finally, with regard to place of residency within the BMA, 62.0% lived in Bucaramanga, 22.7% in Floridablanca, 8.2% in Girón, and 7.1% in Piedecuesta.

**Table 2:**
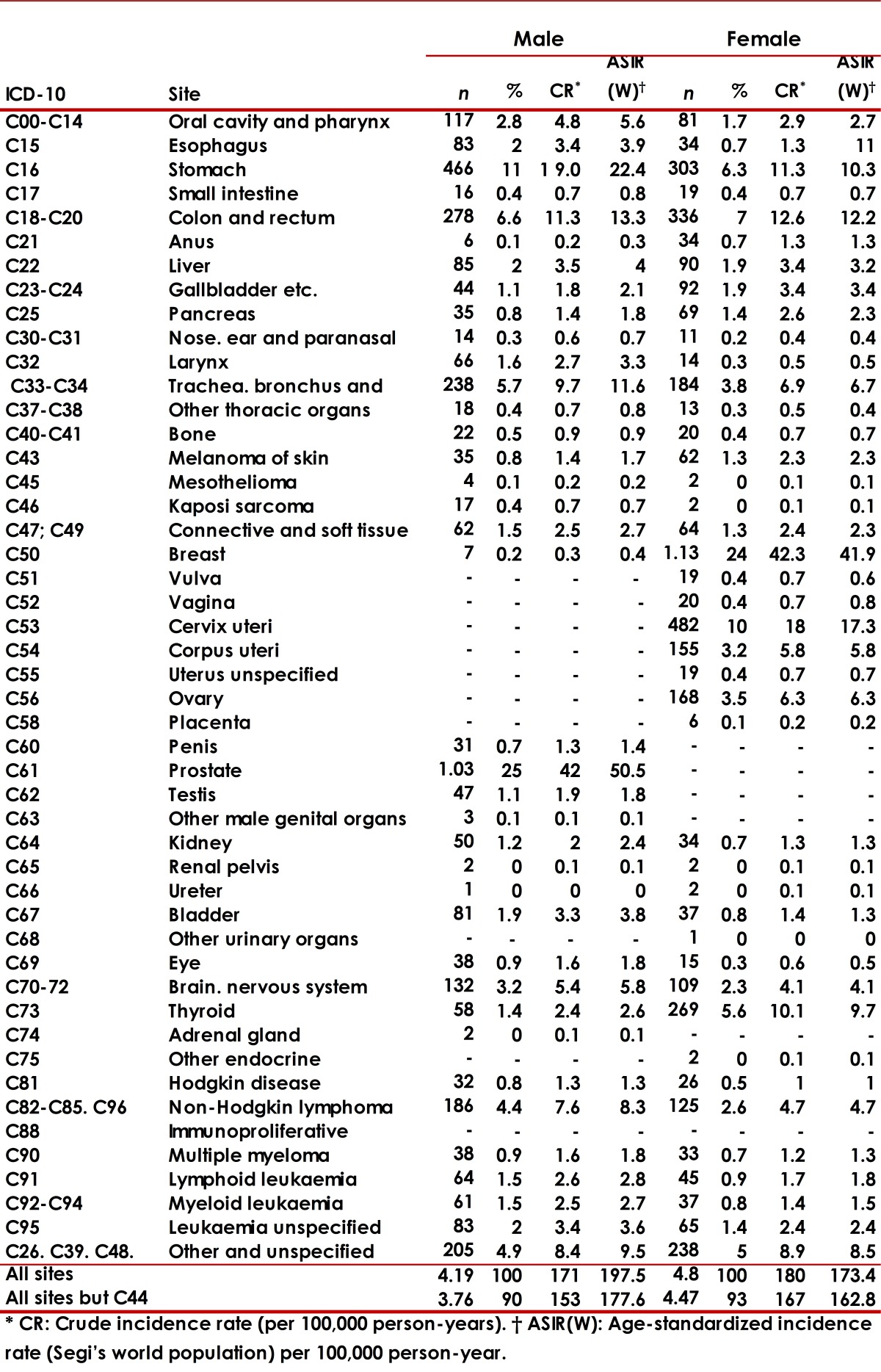
Number of cases, crude and age-standardized incidence rate (Segi's world population) per 100,000 person-years, by location and gender. Bucaramanga Metropolitan Area, 2003-2007.

### Incidence Rates:

**Figure 2:**
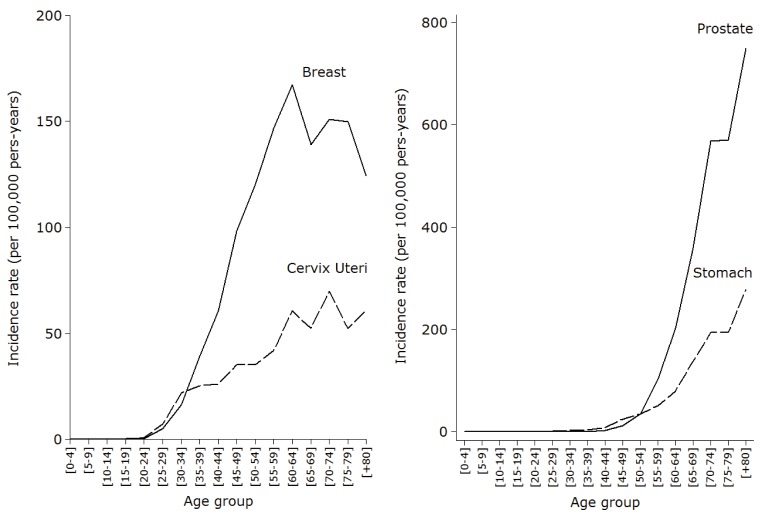
Incidence rate (crude) of the most common types of cancer among women and men resident in the BMA, by age groups, during 2003-2007. Left: Incidence rate (crude) of breast and cervix cancer among women. Right: Incidence rate (crude) of prostate and stomach cancer among men.

ASIR(W) for all cancers, excluding non-melanoma skin cancer, was 162.8 per 100,000 person-years in women and 177.6 per 100,000 person-years in men. In women, the types of malignant tumors with the highest incidence rates were breast (41.9 per 100,000 person-years), cervix (17.3 per 100,000 person-years), colorectal (12.2 per 100,000 person-years), stomach (10.3 per 100,000 person -year) and thyroid (9.7 per 100,000 person-years) (Table 2). In men, the highest incidence rates were reported for prostate cancer (50.5 per 100,000 person-years), stomach (22.4 per 100,000 person-years), colorectal (13.3 per 100,000 person-years), lung (11.6 per 100,000 person-years) and lymphoma (9.6 per 100,000 person-years).Crude incidence rates for the two most common cancers among women showed progressive increases with age that began around the second decade of life (figure 2), however, unlike cervical cancer, whose incidence increased steadily, breast cancer reached a peak incidence in women aged 60-64 years, showing then a downward trend. Regarding the behavior of crude incidence rates in men, although prostate cancer and stomach cancer showed sustained increases with age (figure 2), the first cases of prostate cancer were registered among individuals 40-45 years old, that is, about two decades later than in gastric cancer.

### Mortality

During 2003-2007, there were 23.869 deaths in the BMA among which 4,725 (19.8%) were attributed to cancer. In the latter group, 2.423 deaths (51.3%) occurred in women. Considering together men and women, two out of three deaths were attributed to cancer in any of the following locations: Stomach (15.2%), lung (10.6%), breast (6.8%), colorectal (6.5%), prostate (6.2%), liver (5.6%), brain (4.3%), cervix (4.2%), pancreas (3.4%) or unknown primary tumors (3.1%).

The average age of death for women was 63.9 years with an ASMR(W) of 84.5 per 100,000 person-years. In this population, breast cancer caused the most deaths (13.1%), followed by stomach cancer (12.5%), lung cancer (8.7%), cervix cancer (8.3%), and colon cancer (6.9%) Table 3. In men, the average age of death was 64.4 years with an overall ASMR(W) of 106.2 per 100,000 person-years. In this group, stomach cancer ranked first as a cause of death (18.1%) followed by prostate cancer (12.7%), lung cancer (12.6%), colon cancer (5.5%) and liver cancer (5.4%).

**Table 3.**
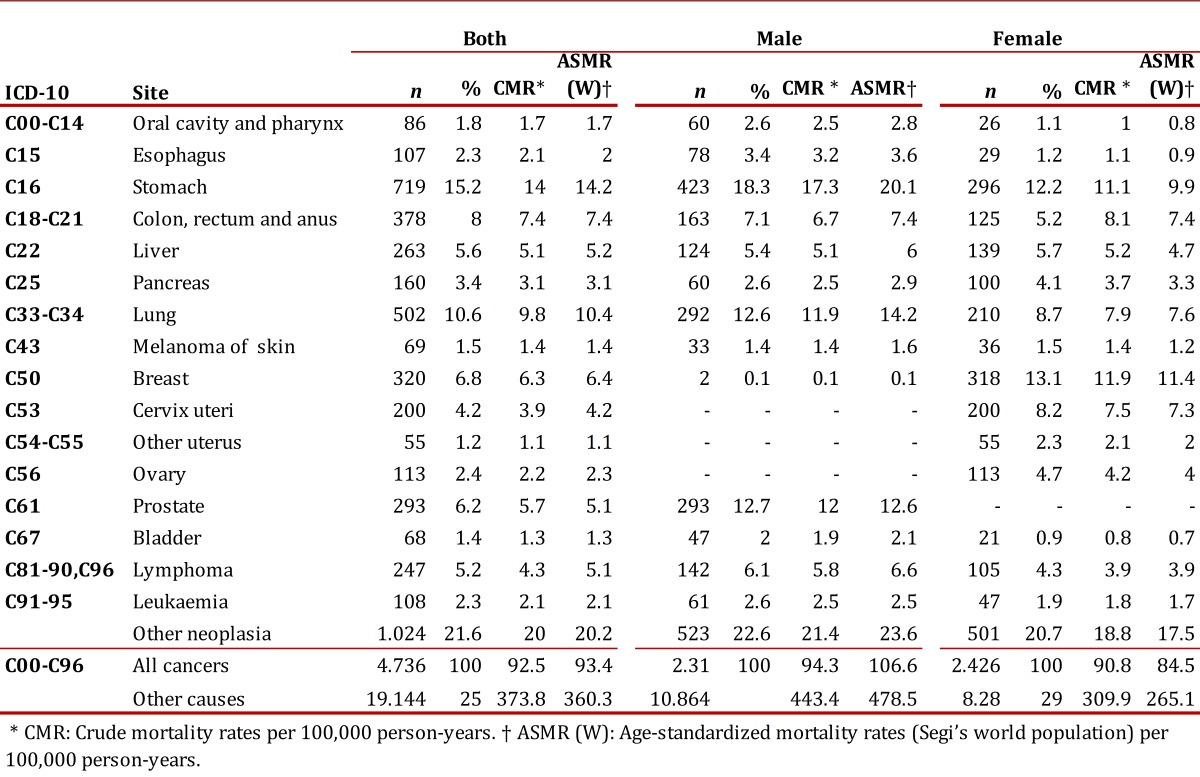
Crude and age-standardized mortality rate (Segi's world population) per 100,000 person-years, by location and gender. Bucaramanga Metropolitan Area, 2003-2007

## Discussion

This report shows the results from the analysis of cancer cases registered by the BMA-PBCR during 2003-2007. Overall, the number of cancer cases reported in women was slightly higher than that reported in men, and 85.5% of all cases had pathological confirmation. The most common cancers reported in women were breast cancer, cervical cancer, and colorectal cancer, whereas the corresponding malignancies in men were prostate cancer, stomach cancer, and colorectal cancer. The ASIR(W) for all cancers, excluding non-melanoma skin cancer, was 162.8 per 100,000 person-years in women and 177.6 per 100,000 person-years in men. Regarding cancer mortality, breast cancer ranked as first cause of deaths among women, followed by stomach and lung cancer. In men, cancer mortality was primarily attributed stomach cancer, followed by prostate and lung cancer.

The degree of data completeness was high. This was achieved in part by the inclusion of cases identified by death certificate only, whose contribution to the totality of the registry data was less than the maximum suggested by the IARC (i.e., between 10-15%)^16^. Deaths certificates are important sources of information that allow identifying some cancers with low pathological detection rates such as cancer of pancreas, lung, ovary, central nervous system and the retinoblastoma. Barriers to the diagnosis and treatment of these highly lethal malignancies may partially explain the limited detection associated to pathological studies.

Regarding the frequency of cancer in the general population, breast cancer ranked first followed closely by prostate cancer. Cervical cancer showed a downward trend between 2000-2004 and 2003-2007 (moved from the third to the fifth place). This finding differs from the national data according to which cervical cancer still ranks second, after breast cancer. On the other hand, in the registry as at the national level, prostate cancer and stomach cancer were the most commonly reported malignancies among men.^17^

Our results showed that men have greater cancer incidence rates than women. Women and men had different profiles regarding the type of cancer affecting them, without considering those types of cancer that are exclusive for each sex. In men from the BMA-PBCR, ASIR(W) for all types of cancer was less than the world and national figures (177.6 per 100,000 person-years versus 204,4 and 186.6 per 100,000 person-years, respectively) ^2^ ^,^ ^17^, and the reference value from the PBCR of Cali (218.2 per 100,000 person-years) . In women from our registry, ASIR(W) was closer to the world figures (162.8 versus 164.9 per 100,000 person-year)^2^ but lower than the national estimates for the 2000-2006 (196.9 per 100,000 person-years)^17^ and the estimates from the PBCR of Cali (196.4 per 100,000 person-years).^18^.Looking at specific sites, in women the ASIR(W) for breast cancer from the BMA-PBCR was similar to the world figures (41.9 versus 39.0 per 100,000 person-years)^2^, higher than the national estimate (36.4 per 100,000 person-years)^17^, and lower than the incidence reported by the PBCR of Cali (48.0 per 100,000 person-years)^18^.Globally, the incidence of breast cancer is increasing, making it the most prevalent malignancy among women currently. In Asia and Latin America cases of breast cancer have increased probably due to the aging of the populations and the increase on the coverage of screening programs, situations that may explain our results^20^.

Regarding to prostate cancer, we observed that the ASIR(W) was well above the worldwide incidence (50.5 versus 28.5 per 100,000 person-years) 2 but similar to the estimate reported by the INC at the national level (47.8 per 100,000 person-years)^17^. These findings may be related to higher screening rates for this cancer in our country that allow detecting early and latent cases. Gastric cancer is also a major problem, particularly among men from our registry (ASIR(W): 22.4 versus 10.3 per 100,000 person-years in men and women, respectively). The same pattern was observed at the national level; however, the incidence rates were consistently higher than ours (26.5 and 15.4 per 100,000 person-years in men and women, respectively).^17^

Estimation of mortality rates allows evaluating the impact of secondary prevention strategies aimed to perform an early diagnosis and improve treatment. In the BMA-PBCR we observed an ASMR(W) of 106.2 and 84.5 per 100,000 person-years in men and women, respectively. Those rates are higher than the corresponding national and departmental figures in the case of men (84.4 and 88.5 per 100,000 person-years, respectively) but lower in the case of women (77.2 and 74.1 per 100,000 person-years, respectively).^17^ ^-^ ^22^.

Taking into consideration the most common tumours in our region (breast, prostate and stomach cancer) we observed that mortality attributable to breast cancer not only ranked first in women (ASMR(W): 11.4 per 100,000 person-years) but also was higher compared to the nation estimate for the period 2002-2006: 9.5 per 100,000 person-years. This finding suggests that the BMA constitutes a geographic area with a high risk of death due to breast cancer and highlights the need of evaluating the screening programs and primary health care offered to the patients^23^. In relation to prostate cancer, it constitutes the second leading cause of death with an estimated ASMR(W) equals to 12.9 per 100,000 person-years. This contrast to the global figures according to which prostate cancer occupies the sixth place among the causes of death attributable to malignancies, without differences between developed and developing countries. Finally, it is also important to consider gastric cancer mortality in the BMA, as it is higher compared to the national figures. This heterogeneity may be due in part to regional differences in screening programs for early diagnosis, considering that this tumour has no specific symptoms at early stages^24^.

## Conclusions

This report demonstrated the magnitude of the problem of cancer in our region and suggested the existence of difficulties in the early detection and timely management programs of this disease. Understanding the problem is just possible by the setting and maintenance of high-quality PBCRs. In general terms, malignant tumour lesions from our registry showed a similar profile compared to that observed in the world population but incidence rates below those estimated for Colombia. Special attention deserves the incidence of breast and prostate cancer whose ASIR(W) surpassed the national rates. Regarding stomach cancer, despite the report of lower ASIR(W) as compared to the rest of the country, ASMR(W) were greater thus indicating that early detection and timely access strategies have to be reviewed. Regarding cancer-related mortality rates, the higher ASMR(W) found in our region as compared to those reported for Colombian is a subject of major concern. Based on our results, reinforcing cancer control measures is essential and a priority. Particularly, it is necessary to focus on primary prevention, effective education to assimilate healthy habits, encouragement to participate actively in good-quality screening programs and the offering of multidisciplinary care based on the protocols of evidenced-based medicine.
